# Physical activity and quality of life predictors among university students with polio in India: A cross-sectional study

**Published:** 2020-08-29

**Authors:** G. Shankar Ganesh, Devashree Marwah, Sukriti Punyal, Sachin Gupta

**Affiliations:** ^1^Department of Physiotherapy, Composite Regional Centre for Skill Development, Rehabilitation, and Empowerment of Persons with Disabilities, Lucknow, Uttar Pradesh, India; ^2^Western Sydney University, Parramatta, Australia; ^3^Safdarjung Hospital, Ansari Nagar East, New Delhi, India; ^4^Jamia Hamdard University, Hamdard Nagar, New Delhi, India

**Keywords:** poliomyelitis, fatigue, limitation of activity, pain sensation, quality of life

## Abstract

**Background:**

Quality of life (QoL) assessments measure the overall well-being of a person. Available data suggest an estimated 10-20 million polio survivors worldwide. Few studies have investigated the physical activity and the QoL of these patients in India.

**Aim:**

The present cross-sectional study attempted to measure the physical activity and QoL of polio survivors and to evaluate the relationship between physical activity and QoL, among other factors.

**Methods:**

The study was conducted in 96 students (64 women and 32 men with a mean age of 22.1±3.7 years). QoL, physical activity, fatigue, and pain were measured by the World Health Organization QoL measure – abbreviated version (WHOQOL-BREF), physical activity scale for individuals with physical disabilities (PASIPD), multidimensional fatigue symptom inventory-short form (MFSI-SF), and numerical rating scale.

**Results:**

The mean metabolic equivalent score was 27.10 h/day. Low mean scores were observed for the physical health, psychological well-being, social relationships, and environmental domains of QoL (25.2±3.3, 21.8±3.0, 12.0±1.8, and 23.0±4.3, respectively). There was a weak negative association between physical activity levels and the physical health domain of QoL (*P*<0.05), whereas no associations were identified between physical activity levels and other QoL domains (P>0.05). Regression analysis identified female gender, fatigue, and physical activity as predictors of the physical health domain, and female gender, number of sites affected, and assistive devices used as predictors of the psychological health domain of QoL.

**Conclusions:**

Low physical activity levels and QoL were noted among students with poliomyelitis in Uttar Pradesh, India.

**Relevance for patients:**

Considering the inverse relationship between physical activity and QoL, physical activities should be modified or protected in polio survivors. Female polio survivors may be referred to psychological counseling to learn coping strategies, even during periods of relative stability.

## 1. Introduction

Poliomyelitis (polio) is caused by a RNA enterovirus in children <5 years of age. Although highly contagious, the majority of these infections is asymptomatic. The virus produces minor non-specific symptoms in only 4%-8% and destroys the motor neurons in a mere 0.5% of infected patients [1]. Data demonstrate that 10%-40% of survivors recover their full muscle strength, whereas the remaining 60%-90% suffer from irreversible asymmetrical motor paralysis ranging from monoplegia to quadriplegia [2]. These varied manifestations of the disability influence the physical health of patients suffering from polio [3]. The effective management of residual polio paralysis requires a multidisciplinary approach comprising physiotherapy, surgery, orthosis, and assistive and ambulatory aids. Polio survivors may develop new disabilities even 30-40 years after the initial infection [4]. “Post-Polio Syndrome” (PPS) refers to the development of new symptoms such as pain, fatigue, and new muscle weakness [5]. Individuals exhibiting substantial recovery after extensive paralysis during active infection [5] and women [6] are at high risk of developing PPS. PPS management mostly focuses on symptom relief [4].

India was among the worst-affected developing nations during the polio outbreak and had the highest number of patients with paralytic polio reported worldwide [7]. Despite being declared polio free by the World Health Organization (WHO) on February 25, 2012 [8], new cases of polio are being reported at endemic rates in India [9]. The available data report an estimated 10-20 million polio survivors worldwide.

Quality of life (QoL) refers to the general well-being of individuals in the context of the culture and value systems in which they live [10]. The QoL must be measured in various populations because traditional measures of health provide limited information of a person’s overall well-being [11]. For instance, no difference in subjective QoL scores was recorded between children and adolescents with or without physical disabilities in Hong Kong [12]. A report from Taiwan [13] exhibited that the QoL in adolescents was not affected by physical disabilities. However, the study suggested that increasing age may have an impact. However, a study in Nigeria reported lower QoL scores in 73 adolescent polio survivors compared to their healthy counterparts [3]. Further, the study exhibited that female polio survivors were significantly more affected than male survivors in the emotional and overall QoL scores. The authors concluded that these results might be attributed to the social isolation and poor integration of children with polio within the Nigerian society. Women polio survivors were also observed to be undereducated and underemployed, thus affecting their QoL [14].

Physical inactivity increases the risk of various diseases such as diabetes, breast and colon cancers, stroke, hypertension, and coronary heart disease [15], whereas regular physical activity reduces the risk of developing chronic diseases and enhances the QoL [16]. Furthermore, literature exhibits that regular physical activity enhances the QoL in older adults [17] and in patients with chronic diseases [18,19]. However, studies report that people with disabilities are less likely to lead a physically active lifestyle compared to their normal counterparts [20,21]. Indians were observed to be more sedentary and less physically active than Caucasians [22], with fewer than 10% of the Indian population engaging in any type of physical activity during their leisure time [23]. A majority of the healthy Indian population also does not meet the global WHO-recommended physical activity intensities [24]. There is a lacuna in scientific literature about the amount of physical activities undertaken by persons with disabilities (PwDs). Further, the influence of stress exerted on the students [25] during education on the amount of physical activity has not been investigated. Studies in developing countries focus on the initial physical disabilities after polio infection [26]. However, literature on the physical activity and QoL of young polio survivors is scarce. The role of ambulatory devices such as wheelchairs and crutches provided under various rehabilitative care programs in influencing the QoL in this population is also scarcely investigated. As the age of the younger population puts additional demand on the health system, the procurement of such information is vital. Meager knowledge is available about polio survivors in the lower- and middle-income countries compared to that in high-income countries. In addition, the existing physical and psychological performance of the polio survivors is relatively unknown until they are diagnosed with PPS.

As the polio endemic in India lasted till 2012, a large population of young polio survivors might develop PPS in the coming years. We hypothesized that the presence of residual disabilities and considerable orthopedic and neurological comorbidities in a majority of patients with polio will affect their level of physical activity, fatigue, and QoL due to the prolonged physical stresses on pre-existing skeletal deformities and weakened muscles. QoL is a complex, subjective, and multidimensional concept that encompasses the physical, psychological, and social well-being of individuals. QoL is influenced by various intrinsic and extrinsic factors. Studies have demonstrated that QoL may be managed by predicting its determinants such as anxiety, depression, fatigue, self-efficacy, and sociodemographic factors such as education, clinical characteristics, and physical activity [27-29]. Evaluating these determinants that may influence the QoL in this population before the onset of PPS would provide a baseline figure for comparing these values with those of patients suffering from PPS. Improving the QoL of and integrating PwDs into the social structure is a major policy goal for all governments. The detailing of this analysis by sociodemographic and clinical characteristics might help to detect small or disease-specific changes, which might assist in determining the most effective ways of control. Therefore, the aim of this study was to determine the physical activity, fatigue level, and QoL of university students affected with polio in Uttar Pradesh, India, and identify the relationship between demographic or clinical data, physical activity, fatigue, pain, and all four dimensions of QoL using the WHO QoL measure abbreviated version (WHOQOL-BREF).

## 2. Materials and Methods

### 2.1. Participants

The present cross-sectional survey was conducted in 96 (32 men and 64 women) full-time students with residual impairments and relative periods of stability following paralytic poliomyelitis to evaluate the physical activity level and QoL of the affected people. The objective of the study and a request for participation were presented to full-time students from our institute and a nearby university located in Uttar Pradesh, India. The specific inclusion criteria were as follows: (i) Established diagnosis of polio, (ii) absence of PPS according to the March of Dimes criteria, 2000 [30], (iii) age group between 18 and 32 years, and (iv) capability to follow instructions related to the questionnaires. The exclusion criteria were as follows: (i) Polio associated with traumatic brain injury, brachial plexus injury, and fractures of the extremities, or other neurological impairments, (ii) presence of any systemic medical disease that would restrict physical activity, (iii) inability to complete the questionnaires, (iv) clinical depression and presence of mental health problems, (v) pregnancy, and (vi) use of medications that might affect the decision-making skills. Attempts were made to exclude participants who showed late effects of polio or symptoms of PPS such as joint and muscle pain, new muscle weakness, increased muscular fatigability, general fatigue, and cold intolerance [31]. The participants were included or excluded based on the recommendation of the general physician and clinical psychologist.

Informed consent was obtained from each participant. Data were collected using questionnaires in combination with interviews to ensure uniformity in the research process. The structured face-to-face interviews lasting an average of 45 min were conducted between January 2017 and June 2018. The participants were given an opportunity to clarify any doubts or questions at the end of each interview.

All participants were able to transport themselves independently, were self-sufficient in everyday life, and did not need any personal assistance in functioning. One of the authors collected the demographic information during the interview, and the participants were provided with instructions on the use and completion of the physical activity scale for individuals with physical disabilities (PASIPD), WHOQOL-BREF questionnaire, and multidimensional fatigue symptom inventory-short form (MFSI-SF) [32].

### 2.2. Variables documented

Demographic characteristics such as age, gender, and education level (certificate, diploma, graduate, postgraduate, or doctoral level) were documented. The age range of 18-32 years was divided into three intervals of 5 years, namely, 18-22 years, 23-27 years, and 28-32 years for convenient data analysis. The polio-specific interviews comprised detailed questions regarding the age of onset of polio and the location of the paralysis. The affected sites were recorded as bilateral lower extremities, single lower extremity, trunk and lower extremities, and upper and lower extremities. Muscle strengths were determined manually according to the Medical Research Council scale for the hip, knee, ankle, shoulder, elbow, wrist, abdominal, and spine extensor muscles. The paralysis location was compared to the sites mentioned in the disability certificate provided by the Government of India and recorded. Patients with discrepancies between the paralysis sites mentioned and the locations identified were excluded. In addition, participants who had developed new weaknesses or disabilities leading to functional deteriorations due to continuous stress on the skeletal deformity and previously weakened muscles were also excluded [33]. The current ambulatory function assistance such as the use of orthoses or walking aids was recorded (wheelchair; tricycle; crutches, orthosis, or assistive devices, motorized wheelers, and the use of multiple walking aids and appliances, if any). The aim was to collect this information during the stable phase of residual paralysis [34].

Physical activity levels were measured using the PASIPD. The PASIPD is a 7-day recall activity questionnaire where the participants are asked to rate their participation in 13 items on leisure, household, and work-related activities as “never,” “seldom (1-2 days/week),” “sometimes (3-4 days/week),” or “often (5-7 days/week).” The number of active hours (1 h, 1-4 h, 5-8 h, and >8 h) per day was recorded as the physical activity levels using the PASIPD. The total score was calculated by multiplying the average hours of participation per day per item and the metabolic equivalent value associated with the intensity of activity. These items were summed together to obtain a final score. The maximum score possible was 199.5 and higher values indicated greater activity levels [35].

The QoL of participants was assessed by the WHOQOL-BREF questionnaire focusing on the individuals’ views of their well-being. The questionnaire comprises 24 items that are divided across the four domains of physical health, psychological health, social relationships, and environmental health. Each item was rated on a scale of 1-5 on the Likert scale. The scores for a participant were excluded when 20% of items or two or more items were missed. The mean score of items within each domain was summed up to calculate the domain score, and the scores transformed on a scale from 0 to 100 [36]. Higher scores indicate higher QoL of the participants.

We used the MFSI-SF to measure fatigue because this scale does not assume the presence of fatigue. The MFSI-SF is a 30-item self-reported, multidimensional measure to assess the various dimensions of fatigue such as general fatigue, physical fatigue, emotional fatigue, mental fatigue, and vigor that the participant experienced in the past 7 days. Each item is rated on a 5-point Likert scale from 0 (not at all) to 4 (extremely). The item scores for each subscale are added to calculate the individual subscale score. Subscale score ranges from 0 to 24. The total fatigue score is calculated by subtracting the vigor subscale score from the sum of general, physical, emotional, and mental subscale scores. The total score ranges from 24 to 96, and higher scores indicate higher levels of fatigue.

The average pain intensity that the participants endured during the past 7 days was quantified using the 11-point numerical rating scale (0 [no pain]-10 [pain as bad as could be]) [37].

### 2.3. Data analysis

The data collected were analyzed using SPSS 20.0 (IBM, Armonk, NY). Basic demographics and clinical characteristics were recorded for all patients. Correlation coefficients were computed between the four domains of WHOQOL-BREF questionnaire and level of physical activity, fatigue, pain, and selected demographic and clinical variables (age, sex, sites affected by polio, and assistive devices used for commuting) using the Pearson product correlation coefficient analysis. Using the Bonferroni approach to control Type I error across the correlations, p < 0.05 denoted significance.

We used dummy variables to sort data (sites affected, assistive devices used, and education) into mutually exclusive categories (such as certificate, undergraduate, postgraduate, and doctoral level for education) for regression analysis. For the two-level gender categorical variable (male and female), we used a dummy variable with value 1 for females and 0 for males. Similarly, for analyzing the sites affected, we used four categorizations (B/L lower extremities, single lower extremity, trunk and extremity, and upper and lower extremities) and required three variables (k-1 dummy variables) in our regression as any one dummy variable was perfectly collinear with remaining set of dummies. For example, when a response was “3,” the other dummy variables would be all 0s. For all analyses, *P*<0.05 was considered statistically significant.

## 3. Results

Out of the 225 students with poliomyelitis who responded to our request, 22 students with complaints of functional deterioration (including development of weakness [less than grade 3] in the trunk muscles of three students) and eight students with complaints of worsening of scoliosis were referred to physicians and orthotists, respectively, for further consultation. Of the total participants, 16 students complaining of severe thoracic pain and 10 complaining of severe lower back pain were excluded and were advised physiotherapy. A total of four students were excluded due to use of antidepressants, and one student was excluded due to a recent fracture of the right forearm. Thus, 164 participants who met the inclusion and exclusion criteria were identified. Of these, 18 participants did not consent to the study due to lack of time, no immediate advantage for participants, and a perceived difficulty in commuting for the study. On receiving the consent, the participants were invited for an interview. Although 140 participants gave consent for the study, only 96 participants (64 women and 32 men; mean±SD age: 22.1+3.7 years) completed the interviews despite repeated follow-up. The age range of the participants was 18-32 years, with a mean±SD age of 22.1±3.7 years. The sites affected by polio and the types of assistive devices used by the participants are presented in Table 1. Of the 96 participants, 6.3% (*n*=6) were enrolled in a certificate or diploma level program, 70.8% (*n*=68) in bachelor degree programs, 18.8% (*n*=18) in postgraduate programs, and 4.2% (*n*=4) in doctoral level programs.

**Table 1:** General characteristics of the study sample.

Age	Sex	Affected sites	Mode of ambulation
22.14	64 F	B/L lower extremities – 44	Wheelchair – 36
(3.69)	32	Single lower extremity – 28	Tricycle – 6
	M	Trunk and extremity – 16	No aids and appliances –16
		Upper and lower extremities – 8	Crutches/calipers/other assistive devices – 20
			Motor two-wheelers – 2
			Multiple appliances – 16

F: Female, M: Male

The interviewed participants demonstrated a mean±SD metabolic equivalent score of 27.1±1.6 h/day of the possible 199.5 metabolic equivalent h/day. In the physical health domain (D1), participants had a mean±SD score of 25.2±3.3. The domains of psychological health (D2), social relationship (D3), and environment (D4) received mean±SD scores of 21.8±3.0, 12.0±1.8, and 23.0±4.3, respectively. The patients reported a mean±SD pain level of 2.5±1.8 on the numerical pain rating scale. The mean±SD fatigue score on MFSI-SF was 17.4±1.7.

**Figure 1:**
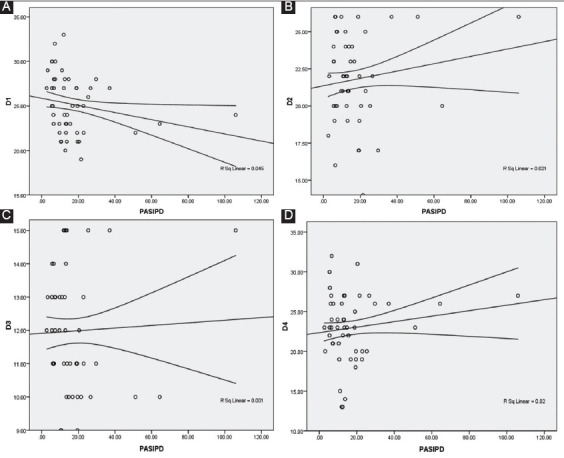
Scatter plots showing the relationship between physical activity and (A) physical health, (B) psychological well-being, (C) social relationships, and (D) environmental domains of QoL among university students with poliomyelitis in Uttar Pradesh, India (*n*=96).

Pearson product correlation coefficient analysis exhibited a weak inverse significant association between the level of physical activity and the physical health domain of QoL (*r*=−0.318, *P*<0.01) (Table 2). There was no association between the levels of physical activity and psychological well-being, social relationships, and environmental domains of QoL (P>0.05), indicating that physical activity did not influence the QoL enjoyed by the participants. A scatter plot of this relationship is presented in Figure 1. A weak inverse association was identified between gender and fatigue, and the physical domain of QoL. Similarly, a weak negative relationship was observed between sex, numbers of sites affected, and assistive devices used, and the psychological domain of QoL. A weak but significant relationship was found between pain and environmental domain of QoL.

**Table 2:** Correlation between quality of life, demographic characteristics, and physical activity among university students with poliomyelitis in Uttar Pradesh, India (*n*=96).

QOL domain	Age	Sex	Education	Sites affected	Assistive devices	Pain	Fatigue	Physical activity
Physical health	−0.061	−0.336**	0.296*	0.132	−0.236	0.123	−0.212*	−0.318**
Psychological health	−0.118	−0.287**	−0.080	−0.271**	−0.195*	0.107	0.145	−0.118
Social relationships	0.123	0.154	0.232	0.022	0.054	0.003	0.038	−0.042
Environment	0.056	0.048	0.036	-0.062	0.152	−0.300**	0.141	−0.073

* *P*<0.05;

** *P*<0.01

Prediction models for multiple regression analysis [38] require 5-20 events per variables for reliable results. Due to the presence of few events per variables, we performed linear regression analysis. The results exhibited that female gender, fatigue, and physical activity statistically predicted the physical health domain (F [3, 92]=8.713, *P*<0.0005, R^2^=0.221), whereas female gender, numbers of sites affected, and assistive devices used predicted the psychological health domain (F [3, 92]=5.497, *P*<0.0005, R^2^=0.152) (Table 3).

**Table 3:** Regression analysis of significant variables predicting quality of life domains among students with polio in Uttar Pradesh, India (*n*=96).

Variable	B	SE B	B	*P*-value
Physical health				
Constant	25.75	1.42		0.00*
Sex	1.241	0.715	0.182	0.08
Fatigue	−0.063	0.018	−0.337	0.001*
Physical activity	−0.036	0.019	−0.189	0.068
Psychological health				
Constant	25.866	1.048		0.00*
Sex	−1.637	0.643	−0.265	0.012*
Sites affected	−0.428	0.313	−0.141	0.175
Mode of ambulation	−0.304	0.127	−0.231	0.019*

* *P* <0.05

## 4. Discussion

In the present study, we evaluated the amount of physical activity and the QoL enjoyed by polio survivors during their periods of stability. The mean physical activity scores reported by the participants were 27.1 h/day, suggesting that patients were physically active for a few hours of the day and few days of the week. However, this score (27.1 h/day) was found to be higher than the scores (18.2 h/day) calculated in Indian patients with paraplegia [11] and those reported by PwDs who considered themselves active or extremely active (22.5 h/day) but lower than those reported (36.3 h/day) by participants who were active in wheelchair basketball [39]. This score was higher than scores reported in other studies conducted by Warms *et al*. [40] (score of 22.5±20.4 h/day) and Washburn *et al*. [35] (score of 20.2±14.5 h/day) that had included patients with post-polio paralysis and other disabilities as respondents.

The average QoL scores in students with polio were observed to be lower than the scores in the normal population in all four domains of the QoL: Physical health (25.2±3.3 vs. 73.5±18.1), psychological well-being (21.8±3.0 vs. 70.6±14.0), social relationships (12.0+1.8 vs. 71.5±18.2), and environmental (23.0±4.3 vs. 75.1±13.0) [41]. These results are concurrent with the findings of On *et al*. [42] who observed that polio survivors without PPS also have lower QoL compared to the normal population. Although other works have also evaluated QoL in polio survivors with and without PPS, the results could not be compared as different instruments were used to evaluate QoL.

Furthermore, the results reported a small but significant negative association between physical activity, sex, and fatigue, and the physical domain of QoL. The regression analysis results exhibited that sex can predict both the physical and psychological domains of QoL. The physical activity and fatigue can predict the physical domain of QoL, whereas the number of sites affected and mode of ambulation used can predict the psychological domain of QoL. These findings are in accordance with the conclusions of a systematic review by Vagetti *et al*. [43] that demonstrated that the association between various domains of QoL and physical activity varies from strong to moderate and inconsistent.

Enhanced QoL is considered a benefit and motivator for physical activity. Although PwDs who enjoyed better QoL scores reported higher physical activity (28.4±30.2 MET h/day) scores compared to those who enjoyed poor and fair QoL (17.9±22.1 MET h/day and 21.7±17.8 MET h/day), the association between physical activity and QoL in PwDs was inconclusive, and physical activity could not explain QoL in this population [44].

A previous study demonstrated that patients with polio were found to be active mostly in household chores for approximately an average of 3 h/day [45]. Winberg *et al*. [46] proposed that factors such as knee muscle strength and gait performance could explain only a small fraction of limitations in physical activity, and the contribution of other factors must be studied in patients with late effects of polio.

It has been postulated that reduced physical activity [47] and social participation [48] can reduce health-related QoL. Studies have exhibited that physical activity improves QoL through exercise, self-efficacy, physical self-esteem, and affects [17,49]. Promoting moderate levels of physical activity among PwDs exhibited improvement in the well-being and prevention of the development of chronic diseases [50]. On the contrary, the results of our study exhibited a weak negative relationship between physical activity and physical health domain of QoL. The physical domain of QoL evaluates pain and discomfort, energy and fatigue, sleep and rest, activities of daily living, dependence on medication and treatment, and ability to work. Although the reasons for this inverse relationship are beyond the scope of this study, we presume that the factors mediating the relationship between physical activity and QoL may play a role. Young adults consider physical self-esteem associated with physical activity to be the main factor, whereas older people believe combined positive and negative effects to influence QoL [17]. Although exercise and self-efficacy were considered an immediate significant mediator between the physical activity and QoL, this association did not maintain significance on follow-up [17,51]. In addition, the dosimetry of physical activity was not examined [35]. It was hypothesized that the association between physical activity and QoL may vary according to the type and severity of the physical activity and different domains of QoL [52]. Patients with polio should consider protecting overused joints and weak muscles before engaging in physical activities [53]. Considering the health benefits of physical activity on PwDs [54], physical activities must be modified, adapted, or provided with additional assistance.

The results exhibited an inverse relationship between the physical domain of QoL and fatigue. Fatigue plays a role in the QoL of patients with PPS [42] and the same appears to be true for polio survivors. The participants had a mean MFSI score of 17.4, which was higher than the fatigue score reported by patients with gynecological cancer (14.4±15.9) [55]. Wheelchair dependency has been related to fatigue in polio survivors with PPS [56]. The high levels of fatigue seen in polio patients may be attributed to emotional factors, central nervous system fatigue, general fatigue, and peripheral neuromuscular fatigue [57]. Efforts must be taken to reduce fatigue in polio by altering lifestyle factors, providing rest periods, and using assistive devices [58].

Education positively influences the physical domain of QoL and has been established as a predictor of QoL across different populations [59-61] due to the high personal control [61,62] it offers. This result is mirrored in other Indian studies that have shown an increase in QoL with increasing education [63,64].

Gender was found to be negatively associated with the psychological and physical domain of QoL. Apart from being a biologically innate trait, gender is considered a set of characteristics that define the role and performance of a person in the community and influences the social expectations attached to it [65]. Other studies have exhibited that polio survivors struggle to maintain the societal activities associated with one’s gender, affecting their self-esteem, physical performance, familial interactions, and health outcomes [66]. Another concern is the risk that PwDs may be treated as a homogenous, asexual, and genderless group.

No significant differences in QoL were identified between both genders in this study. Males had a mean score of 26.6, 20.6, 12.4, and 23.2, whereas females exhibited a score of 24.4, 22.4, 11.8, and 22.8 for the domains of physical health, psychological well-being, social relationships, and environmental domains, respectively. This may also be interpreted as the absence of identified impairments, with factors unrelated to true disability such as stature and strength playing a role. In our study, we observed that females exhibited lower QoL in the physical health and psychological domains. The reason for this finding may be that women report higher number of disabilities compared to men and live for more years with disability [67].

The present study demonstrated that the number of sites affected by polio can predict the psychological domain of the QoL. Studies have exhibited that paralytic involvement of all four limbs can pose new health problems in polio survivors [68], and polio survivors suffer from higher rates of respiratory, cardiac, musculoskeletal, and gastrointestinal system disorders [69]. The present study exhibited an inverse relationship between assistive devices and the psychological domain of QoL. Shiri *et al*. [70] evaluated the psychological health with long-standing poliomyelitis, with or without PPS, demonstrating that emotional distress was higher in the population with polio compared to the normal population. This higher emotional disturbance was not associated with the functioning level of polio patients. The study also demonstrated no difference in the perception of general health between polio participants with or without PPS. Literature has shown that the domain of psychological functioning can influence other domains in the QoL for all disorders [71]. The sense of well-being is a multidimensional concept [72]. Mobility limitations are only one aspect of that dimension and can reduce positive effects [73,74]. Studies have exhibited that relation between functional limitations, use of assistive devices, and well-being is complex and that assistive devices do not always improve a person’s well-being [75,76].

Although studies have attempted to determine factors influencing QoL in PwDs [77,78], the results are inconsistent due to the largely subjective concepts of QoL measures [79]. Rajati *et al*. [27] conducted a cross-sectional study to evaluate the predictive role of the demographics, psychological factors, clinical characteristics, and physical activities using the 36-item short form health survey (SF-36) among 302 PwDs. The results of the study exhibited that gender, self-reported physical activity levels, use of the disability aid tools, and depression were able to predict the variations in physical activity summary. The study also demonstrated that anxiety, depression, and self-efficacy are significantly associated with mental component summary variations.

PwDs experience difficulty in undertaking various activities of daily living. However, not enough information is available regarding the effect of various health and social security services offered to this population for meeting their needs [80], and polio survivors are often left to face challenges on their own. Thus, clinicians should routinely assess the QoL of polio survivors at different intervals. Further qualitative studies and focus group interviews are required to identify the reasons for the low QoL expressed by this study sample. Additional studies should be conducted comparing physical activity and QoL in polio survivors with and without symptoms of PPS.

The present study has several limitations. One of the potential limitations of this study was that the number of the women participants outnumbered the male participants. This might have influenced the effect of the gender on the QoL (physical and psychological domains). Furthermore, we did not collect other information such as body mass index, presence of dyspnea, and the effects of previous surgeries. It is also not known if the participants required personal assistance for other vocational activities and if the participants had to switch their use of assistive devices from one device to another. Because the study was restricted to participants who were enrolled in university, the influence of the stress of education on the participants is not known. Further, it is also possible that those individuals who receive education are likely to have several advantages such as personal independence, community integration, and employment apart from other social, physical, and psychological benefits. The results of this study may not be extrapolated to polio survivors not enrolled in higher education. Other problems associated with polio such as the role of family, history of falls, and balance issues were not considered. The assessment of the QoL and physical activity refers to the level of function at the time of the assessment only. The purpose of measuring QoL itself is a limitation as the rating is subjective and is based on the personal life expectations of the polio survivor. Further, as polio is a childhood disability, the degrees of adjustment undertaken and the participant’s experience with existing health/rehabilitation system could affect their perception about QoL. However, the present study suited its objective by identifying the association between QoL and other variables in individuals with polio in the developing world.

## 5. Conclusions

The overall QoL and physical activity level are low in students affected with polio. Rehabilitation of individuals affected with polio should focus on all aspects of QoL domains. Weak inverse association between the physical activity and physical health domain of QoL has been identified in this study. Considering these findings, we suggest caution and protection when increasing physical activity in this population. As female gender is more likely to be affected by the disease, coping strategies and psychological counseling may be added as part of rehabilitation of polio survivors.
